# Eyelash Epilation in the Absence of Trichiasis: Results of a Population-Based Prevalence Survey in the Western Division of Fiji

**DOI:** 10.1371/journal.pntd.0005277

**Published:** 2017-01-23

**Authors:** Colin Macleod, Chelsea Yalen, Robert Butcher, Umesh Mudaliar, Kinisimere Natutusau, Mere Rainima-Qaniuci, Chris Haffenden, Conall Watson, Naomi Cocks, Luisa Cikamatana, Chrissy H. Roberts, Michael Marks, Eric Rafai, David C. W. Mabey, Mike Kama, Anthony W. Solomon

**Affiliations:** 1 Clinical Research Department, London School of Hygiene & Tropical Medicine, London, United Kingdom; 2 Ophthalmology Department, Lautoka Hospital, Lautoka, Fiji; 3 World Health Organisation, Fiji Country Office, Suva, Fiji; 4 Department of the History of Science and Ideas, Uppsala University, Uppsala, Sweden; 5 Department of Infectious Disease Epidemiology, London School of Hygiene & Tropical Medicine, London, United Kingdom; 6 Department of Communicable Diseases, Ministry of Health, Suva, Fiji; 7 Hospital for Tropical Diseases, London, United Kingdom; Fondation Raoul Follereau, FRANCE

## Abstract

**Background:**

The WHO definition of trachomatous trichiasis (TT) is “at least one eyelash touching the globe, or evidence of recent epilation of in-turned eyelashes”, reflecting the fact that epilation is used as a self-management tool for TT. In Fiji’s Western Division, a high TT prevalence (8.7% in those aged ≥15 years) was reported in a 2012 survey, yet a 2013 survey found no TT and Fijian ophthalmologists rarely see TT cases. Local anecdote suggests that eyelash epilation is a common behaviour, even in the absence of trichiasis. Epilators may have been identified as TT cases in previous surveys.

**Methods:**

We used a preliminary focus group to design an interview questionnaire, and subsequently conducted a population-based prevalence survey to estimate the prevalence of epilation in the absence of trichiasis, and factors associated with this behaviour, in the Western Division of Fiji.

**Results:**

We sampled 695 individuals aged ≥15 years from a total of 457 households in 23 villages. 125 participants (18%) reported epilating their eyelashes at least once within the past year. Photographs were obtained of the eyes of 121/125 (97%) individuals who epilated, and subsequent analysis by an experienced trachoma grader found no cases of trachomatous conjunctival scarring or trichiasis. The age- and sex- adjusted prevalence of epilation in those aged ≥15 years was 8.6% (95% CI 5.7–11.3%). iTaukei ethnicity, female gender, and a higher frequency of drinking kava root were independently associated with epilation.

**Conclusion:**

Epilation occurs in this population in the absence of trichiasis, with sufficient frequency to have markedly inflated previous estimates of local TT prevalence. Individuals with epilated eyelashes should be confirmed as having epilated in-turned eyelashes in an eye with scarring of the conjunctiva before being counted as cases of TT.

## Author Summary

Trachoma, caused by infection with ocular strains of *Chlamydia trachomatis*, represents a major public health issue, and is targeted for global elimination as a public health problem by the year 2020. Until recently, data on trachoma in the Pacific Island states have been sparse, with marked variability in the findings of surveys. The most recent studies in Fiji’s Western Division were conflicted in their estimates of the prevalence of the advanced, blinding, stage of the disease known as trachomatous trichiasis or “TT”. TT results from repeated bouts of infection and resolution, leading to scarring of the eyelid tissue, which causes in-turning of the eyelashes in some individuals so that they grow to touch the globe of the eye. In populations without ready access to healthcare services, individuals may try to self-manage TT by epilating their eyelashes, so that the classical trichiasis sign of contact between eyelashes and eyeball is not seen in surveys. Therefore, the World Health Organization (WHO) definition of TT includes “evidence of recent epilation of in-turned eyelashes”. In the Western Division of Fiji, we carried out a population-based prevalence survey to estimate the prevalence of this behaviour, and to examine associated risk factors. The estimated population prevalence of epilation was 8.6% of those aged ≥15 years, consistent with previously reported estimates of TT in this population, and, importantly, was not associated with any other evidence of advanced trachoma. These data suggest that eyelash epilation is common here, and could inflate estimates of TT wherever such a custom is common. In trachoma surveys, trachomatous scarring should be confirmed to be present when reporting the presence of TT.

## Introduction

Trachoma is the leading infectious cause of blindness globally, responsible for the irreversible loss of vision in 1.9 million people.[1] It is caused by repeated bouts of conjunctival *Chlamydia trachomatis* infection and resolution during childhood, resulting in the gradual accumulation of trachomatous conjunctival scarring (TS). Scarring may progress to distortion of the eyelid and in-turning of the eyelashes to the point that they touch the eyeball (trachomatous trichiasis, TT). Abrasion of the cornea can lead to opacity and blindness[2].

There is international commitment to the global elimination of trachoma as a public health problem by 2020, defined as a reduction in the prevalence of TT unknown to the health system in adults aged ≥15 years to <0.2%, and a reduction in the prevalence of the active trachoma sign trachomatous inflammation—follicular in 1–9 year-olds to <5% [3], by means of the SAFE strategy[4]: Surgery for TT, Antibiotics to clear infection, Facial cleanliness and Environmental improvement to reduce transmission[5]. Accurate estimates of the prevalence of TT are crucial for intervention planning and monitoring progress towards elimination.

TT is an irritating, painful condition that causes significant morbidity to affected individuals.[6,7] Those afflicted may find relief by epilation, which is a traditional treatment for TT in some settings. Epilation can lead to reasonable outcomes where surgery is unavailable, delayed or refused, particularly if caregivers are trained and equipped to do it well[8,9]. To acknowledge that this practice occurs and that if not recognised can obscure the presence of TT, the definition of TT in the WHO simplified trachoma grading system is “at least one eyelash rubs on the eyeball, or evidence of recent removal of in-turned eyelashes”[10].

The extent to which eyelash epilation in the absence of trichiasis can affect TT estimates in trachoma surveys is previously unstudied. Due to the low TT prevalence threshold required to declare elimination of trachoma, significant sources of false-positive TT diagnoses need to be identified. A small number of false positive TT cases recorded by survey teams, when extrapolated to population-level estimates, could lead to the unnecessary training of many trichiasis surgeons. This would be an unnecessary expense and needlessly divert trained healthcare personnel from their regular duties, in places where healthcare staff in general are often in short supply.

Fiji is an archipelago of over 300 islands in the South Pacific, divided geographically into four administrative divisions: Central, Northern, Eastern and Western, with a combined population of approximately 837,300 people.[11] Recent surveys have indicated that trachoma is endemic in Fiji, although prevalence estimates of TT have varied widely. A trachoma rapid assessment in 2007 found 59/313 (19%) of adults over the age of 40 years living in suspected high-risk areas to have evidence of TS, but did not find any cases of TT[12]. A population-based prevalence survey conducted in 2012 identified almost 150 people with TT, and estimated the population prevalence in the Western Division to be 8.7%, among the highest in the world[13]. In response to these results, a second population-based prevalence survey, supported by the Global Trachoma Mapping Project, was carried out in Fiji’s Western Division in 2013 in order to re-estimate the prevalence of signs of trachoma and of conjunctival *C*. *trachomatis* infection. It found no cases of trichiasis in a study population of 2306 people in 31 communities: an estimated prevalence of trichiasis of 0%[14]. During that study, we heard local anecdotes that suggested some iTaukei Fijians may practice epilation in the absence of TT symptoms, as a sociocultural behaviour. These individuals might be incorrectly graded as having TT: an individual cannot truly have trichiasis if the epilated eyelashes are not in-turned. This could have major implications for trichiasis estimates and surgery planning in suspected trachoma-endemic populations in which such behaviour is common.

We sought to understand the motivations for and significance of eyelash epilation in Western Division by first convening a focus group of individuals who reported regularly practising this behaviour. Using their responses to design a questionnaire, we conducted a population-based prevalence survey to estimate the prevalence of epilation, and factors associated with it, in the Western Division of Fiji.

## Methods

### Focus group discussion

The initial focus group discussion was conducted in 2013 alongside a population-based trachoma prevalence survey[14]. We worked in one iTaukei Fijian village in which adults were known to epilate, and following consultation with the village chief, identified adults who had epilated at least every 3 months for at least 1 year, using a snowball sampling approach[15]. Informed written consent was obtained from each participant. A focus group discussion was convened and open questions were used to draw out each individual’s perceptions of factors associated with epilating behaviour. Each participant’s eyes were examined for clinical signs of trachoma by a Global Trachoma Mapping Project-certified grader[16] according to the WHO simplified trachoma grading scheme[10], with photographs of the upper eyelid in primary position, and of the conjunctiva taken to allow later independent review.

The focus group moderator used a list of questions (S1 Appendix) to guide discussion about existing knowledge of trichiasis and epilation, and the causes and natural history of common local eye complaints. A Fijian eyecare nurse assisted with the discussion to clarify cultural nuances, and to provide translation if needed. Participants were encouraged to express themselves in the language in which they were most comfortable. The discussion was audiotaped and transcribed as soon as possible after the event, with input from a Fijian translator where necessary.

### Questionnaire development

The transcript was analysed independently by two researchers (CM & RB) using conventional content analysis, with the coding unit as an idea or phrase within a sentence, and each unit classified into constituent themes. From the themes derived, we developed a questionnaire for the population-level survey. Variables assessed included basic demographics, kava drinking habits, ophthalmic history, details of eyelash epilation, and symptoms or factors which influenced the decision to epilate (S2 Appendix). The questionnaire was designed for use with the Open-Data-Kit Collect (https://opendatakit.org) survey data collection platform for Android smartphones.[17,18]

### Prevalence survey study design

The epilation prevalence survey was conducted in 2015. A cross-sectional cluster random sampling methodology was employed to select individuals aged ≥15 years in the Western Division of Fiji, with villages or settlements used as the primary sampling unit (cluster) and the household used as the secondary sampling unit. Clusters were selected randomly from a list of all non-urban communities in the Western Division using a probability proportional to size methodology. Rural communities in Fiji commonly have one majority ethnicity, and the organisational structures between ethnic groups are distinct and easily identifiable.

Assuming a design effect of 2, we estimated 768 adults over the age of 15 years would be needed to have 95% confidence of detecting a community prevalence of eyelash epilation in adults of 10% with a precision of +/- 3%. Based on the number of available adults per household in previous surveys and assuming 30 households would be surveyed per day, we estimated the required sample size would be achieved from 26 clusters.

### Sampling and examination

In selected clusters, households were randomly selected from a list created on the day of survey in collaboration with local headmen, village chiefs, or healthcare workers. Following consent from the head of household, all those aged ≥15 years resident in selected households were invited to participate. Consenting participants were interviewed individually in their households. Questionnaire responses were recorded electronically in the smartphone application. Those who reported epilating their eyelashes at least once within the past year were invited for ocular examination and photographs of the upper eyelid in primary position, and of the conjunctiva. Consenting participants’ eyes were examined and graded using the WHO simplified trachoma grading system by field workers trained in trachoma grading.[10] Photographs were collected according to a standardised protocol[19] using a Nikon D60 SLR camera with specialised macro lens to allow retrospective grading of the clinical findings.

### Ethical clearance and consent

Ethical approval was obtained from the research ethics committees at the London School of Hygiene & Tropical Medicine (reference numbers 012–354 & 9621) and the Fijian Ministry of Health and Medical Services. Local health workers were contacted in advance of the survey to allow community sensitisation. Survey teams engaged in sevu-sevu (a traditional gift of kava roots) with village leaders where appropriate. Written informed consent was obtained from all participants, with a thumbprint considered acceptable in those unable to provide a signature. Information sheets and consent forms were provided in English and Fijian language, and the local nurse provided translation where needed. Participants were advised that they could withdraw from the survey at any time without adverse consequence to them. A parent or guardian provided informed consent and was always present for those aged 15–17 years as well as individuals with mental or physical disabilities. Any individuals found to have ocular pathologies were referred to the nearest eyecare centre using a standard referral form. All data were anonymised and stored on a secure cloud-based server.

### Data analysis

Photographs were independently graded by two experienced trachoma graders masked to the clinical assessments. Prevalence estimates were adjusted for age and sex using the 2007 census of Fiji.[11] Confidence intervals were calculated by bootstrapping adjusted cluster-level estimates. A two-level random-effects logistic regression analysis was performed to create a causal risk factor model, against the binary outcome of the presence or absence of the behaviour of eyelash epilation at individual level, accounting for clustering at household- and cluster-level. Variables were assessed for collinearity by tabulation and evaluation with a Mantel-Haenszel Chi^2^ test. Variables statistically significant at the p = 0.10 level (Wald’s test) on univariable analysis were considered for the multivariable model. Variables in the multivariable model were assessed by stepwise inclusion, with factors retained in the model if they reached significance at the p<0.05 level (Likelihood ratio test). Data were analysed using Stata version 13.1 (StataCorp, College Station, TX, USA).

## Results

### Focus group discussion

The focus group was carried out in October 2013. Focus group participants were 6 iTaukei Fijians inhabiting one village in the southern part of the Western Division of Fiji. The group was composed of 2 male construction labourers and 4 female housewives with a median age of 42 years [range 20–53 years]. The village where the focus group occurred was considered by residents to be of wholly iTaukei Fijian ethnicity. No participants were found to have any sign of trachoma on clinical examination.

### Transcript analysis

**Fig 1:**
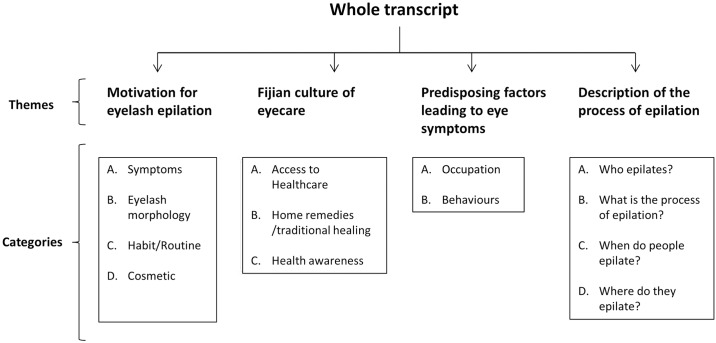
Categories and themes derived from the transcript of the focus group of epilating individuals, Western Division, Fiji, 2013.

The points discussed during the session fell into 4 themes: motivation for epilation, perceived predispositions to eye symptoms, the Fijian eyecare culture, and a description of the epilation process. The themes and categories derived from the data are shown in Fig 1.

Several themes were identified as perceived motivations for eyelash epilation. In each case, transcript extracts have been provided to support the extracted theme. The most commonly cited symptom that could be relieved by epilation was itchiness.

- “When it’s itchy, I’ll start pulling the hair out”.

The group felt that eyelashes targeted for epilation were abnormal, being short and sharp and able to be removed painlessly.

- “the eyelashes that we pull out, they are different from the normal one.”
- “they are prickly. It’s only seen from pigs, swines, they have straight, short, sharp eyelashes”.

The group described a traditional method of “threading” using fibres from coconut husks, the technique for which was demonstrated during the discussion.

- “They use a coconut husk like that (demonstrates)”
- “And they don’t use the pluckers (tweezers)”

Occasionally people would take out large numbers of eyelashes at one time:

- “I pulled out about 120 from my eyes, both of them, with the small ones. Only that was the first time I did that”

Some participants felt that epilation was a habit, but others felt it was more a routine part of their culture.

- “We check our eyes after two, three months…, then when you start with it, then it does become a habit”
- “it’s a habit, once you start you can’t stop so you try to get it out every time”

The participants described good access to and engagement with healthcare services through pharmacists or hospitals when deemed appropriate.

- “we’re educated now, we’re trying to go to the hospitals. Before they did not go to the hospitals.”

Participants described eyecare home remedies and long-held practices

- “actually that was our old people that has been sent from this generation to that generation downwards. It has been, from that time till now we do that for pain relief”
- “In some villages we Fijians, we use herbs. If someone has an eye problem or so he gets these and then we just drop them into the eye and then off they go”.

Participants believed epilation-inducing symptoms could be precipitated by occupational exposures, such as sunlight and dust.

- “(it can be brought on by) not using safety goggles and working out in the hot sun, and then out on the roadside”.

Some felt that symptoms were associated with the iTaukei cultural practice of drinking kava, as well as the long nights associated with its consumption

- “one thing is kava, drinking kava, you have more time drinking of kava and less time sleeping.”

### Prevalence survey

The population-based survey was carried out from July-September 2015. 695 participants aged ≥15 years from 457 households in 23 clusters consented to participate. Three clusters could not be reached due to logistical issues at the time of survey. 16 clusters were considered to be of iTaukei ethnicity, 3 clusters were considered Indo-Fijian ethnicity, and 4 clusters did not have a clear majority ethnic group. 512 of 695 (74%) participants were female. 437 (63%) participants were ethnic iTaukei and 235 (34%) participants were ethnic Indo-Fijian. The median age of study participants was 43 years (IQR 30–56; total range 15–88).

125 (18%) of the 695 individuals interviewed reported epilating their eyelashes at least once within the past year. The overall sex- and age-adjusted prevalence estimate of epilation behaviour in those aged ≥15 years in the Western Division was 8.6% (95% CI 5.7–11.3%).

**Fig 2:**
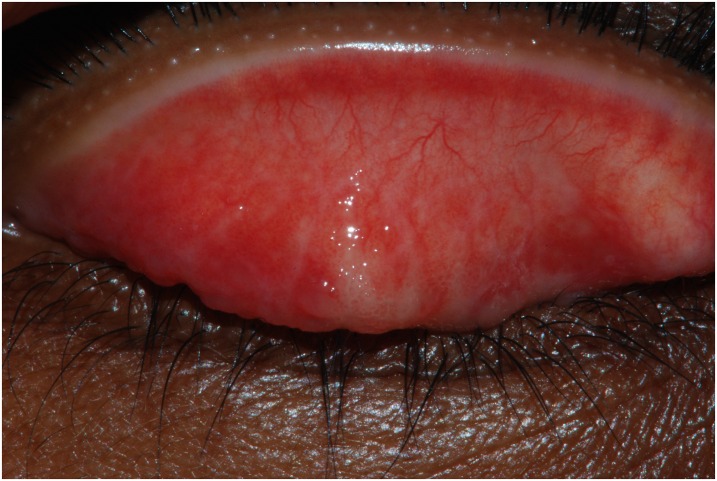
Conjunctival photograph of the individual felt to demonstrate equivocal evidence of conjunctival scar, Western Division, Fiji, 2015.

Of the 125 individuals reporting epilation, 124 (99%) consented to examination and conjunctival photography. In 4 of 124 (3%) consenting participants, one or both eyelids were unable to be everted due to discomfort. On examination, no cases of trichiasis were identified, but one suspected case of TS was identified. On subsequent analysis of conjunctival photographs by two independent trachoma graders, no cases of trichiasis or definite TS were identified, with the one clinically suspected TS case thought to have (at best) equivocal evidence of conjunctival scar (Fig 2). Therefore, either none or only one of those who epilated had any evidence of cicatricial trachoma (TS or TT).

### Description of epilation habits

**Fig 3:**
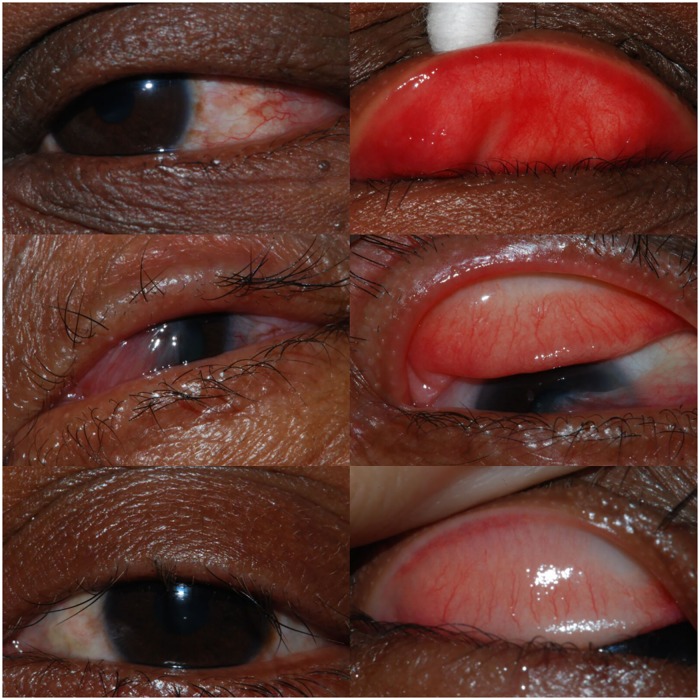
Front-of-eye and conjunctival photographs of individuals who removed (Bottom) 2–10 eyelashes, (Middle) >10 eyelashes (with pterygium), or (Top) most or all of the eyelashes; Western Division, Fiji, 2015.

Data on the 125 epilators are shown in Table 1. The most commonly reported reason for epilating was eye itchiness (111/ 125 epilators, 89%). 80 (64%) reported that they removed >10 eyelashes each time they epilated, and 80 (64%) reported that they epilated on average every 1–3 months. Front of eye and everted lid photographs from epilators with varying frequencies of epilation behaviour are shown in Fig 3.

**Table 1:** Characteristics of eyelash epilation among 125 epilators, Western Division, Fiji, 2015.

Description of epilation	n(%)^a^
Number of lashes removed	
Most or all	3(2.4)
>10	80(64.0)
2–10	40(32.0)
1	2(1.6)
Frequency of epilation	
At least weekly	4(3.2)
At least monthly	16(12.8)
Every 1–3 months	80(64.0)
Less than every 3 months	25(20.0)
Median age at first epilation (years)	29(IQR 20–36)
Reasons for epilating	
Itchiness	111(88.8)
Feeling of dust	76(60.8)
Habit	54(3.2)
Abnormal eyelash	37(29.6)
Cosmetic reasons	4(3.2)
Tradition	0(0.0)

^**a**^ Total number of self-reported eyelash epilators = 125

### Risk factor analysis

Table 2 shows the univariable analysis of each potential risk factor against the outcome of eyelash epilation behaviour. Responses relating to a history of watery eye or eye discharge in the previous week were omitted from analyses because of translation difficulties during the survey.

**Table 2:** Univariable multi-level random effects logistic regression model for associations of eyelash epilation, Western Division, Fiji, 2015.

Variable		Total (%)	Number of epilators (%)	Odds Ratio	95% Confidence Interval	p-value^a^
Ethnicity	Indo-Fijian	235 (33.8)	7 (5.6)	1	-	-
	iTaukei	437 (62.9)	117 (93.6)	10.7	4.6–25.1	<0.001
	Other^b^	23 (3.3)	1 (0.8)	1.8	0.2–15.9	0.615
Gender	Male	183 (26.3)	14 (11.2)	1	-	-
	Female	512 (73.7)	111 (88.8)	2.9	1.6–5.3	0.001
Age (years)	15–24	93 (13.4)	22 (17.6)	3.4	1.7–7.1	0.042
	25–34	154 (22.2)	33 (26.4)	2.5	1.3–4.8	
	35–44	118 (17.0)	23 (18.4)	2.0	1.0–4.1	
	45–54	136 (19.6)	24 (19.2)	2.0	1.0–4.0	
	55+	194 (28.0)	23 (18.4)	1	-	
Frequency of kava drinking	Daily	38(5.5)	8(6.4)	2.1	0.8–5.2	*0*.*0608*
	At least weekly	137 (19.7)	33(26.4)	1.8	1.0–3.0	
	More than monthly but less than weekly	82 (11.8)	22 (17.6)	1.8	0.9–3.3	
	Less than monthly	39 (5.6)	6 (4.8)	0.7	0.3–1.8	
	Never	399(57.4)	56(44.8)	1		
Eye itchiness in the previous week	No	406 (58.4)	34 (27.2)	1	-	-
	Yes	289 (41.6)	91 (72.8)	4.5	2.8–7.1	<0.001
Eye redness in the previous week	No	492 (70.8)	72 (57.6)	1	-	-
	Yes	203 (29.2)	53 (42.4)	1.7	1.1–2.7	0.015
Past ophthalmic history	None	611(87.9)	120(96.0)	1		0.6737
	Blind	2(0.3)	0(0.0)	-		
	Current Cataract	12(1.7)	3(2.4)	1.2	0.3–4.9	
	Previous cataract surgery	4(0.6)	0(0.0)	-		
	Treated for active trachoma	2(0.3)	1(0.8)	2.4	0.1–38.7	
	Traumatic ocular injury	4(0.6)	1(0.8)	2.0	0.2–23.5	
**Total**		**695**	**125**	**-**	**-**	**-**

^a^ Wald’s test

^b^ European, Chinese, Rotuman, Other Pacific islanders

### Multivariable model

Results of the two-level multivariable model are shown in Table 3. In the full model, being a regular epilator was associated with being iTaukei (rather than any other ethnicity) (OR 6.0 95%CI 2.6–13.9), and female gender (OR 4.1 95%CI 2.0–8.6). In addition, those who epilated had a higher odds of reporting being a kava drinker (OR 1.7 95%CI 1.1–2.7).

**Table 3:** Multivariable multi-level random effects logistic regression model for associations of eyelash epilation, Western Division, Fiji, 2015.

Variable	Adjusted Odds Ratio	95% Confidence interval	p-value^a^
Female sex	4.1	1.9–8.0	<0.0001
iTaukei ethnicity ^b^	6	3.0–18.6	<0.0001
Kava drinking frequency			
Daily	4.5	1.6–14.6	0.0096
At least weekly	1.9	1.1–3.5	
At least monthly	2.2	1.0–3.6	
Less than monthly, or never	1	-	

^a^ Likelihood ratio test

^b^ Compared to Indo-Fijian, European, Chinese, Rotuman, Other Pacific islanders

When the reported frequency of kava drinking was considered in the full model, a higher frequency was independently associated with increased odds of being a regular epilator, with those reporting drinking kava daily having odds 4.9 times higher than those who reported drinking kava less than monthly or not at all (OR 4.9, 95%CI 1.6–15.2).

Despite being significant on univariable analysis, age was not associated with regular epilation in the final model. Of note, the effect of age was markedly decreased when the frequency of kava drinking was included in the model, suggesting that this was in part explained by an increased frequency of kava drinking in younger participants. Eye itchiness in the preceding week was strongly associated with being an epilator on univariable analysis (OR 4.5 95%CI 2.8–7.1), but was not included in the final model as this was collinear with kava drinking.

The effect of the frequency of kava drinking on epilation was not confounded by ethnicity, and there was no evidence of interaction between ethnicity and kava drinking frequency on epilation (Likelihood Ratio Test, p = 0.44).

## Discussion

We have documented the presence of a common behaviour in Fiji that has not previously been described in the literature. Eyelash epilation in the presence of the distorted eyelid morphology associated with cicatricial trichiasis is a frequent finding in trachoma-endemic populations[9,20–22]. By contrast, individuals in both phases of this study carried out regular epilation in an area where little, if any, trichiasis is found. According to the WHO simplified trachoma grading system, evidence of recent removal of in-turned lashes should be graded as TT. However, it is difficult to provide guidance on how a grader should determine whether an already-epilated lash was misdirected while it was still in situ. Additionally, in the simplified grading scheme, the conjunctiva does not need to be examined to assign a grade of TT. According to the current system, then, individuals who epilate in-turned eyelashes would be correctly graded as having TT even if they have trichiasis for reasons unrelated to trachoma. It is possible, therefore, that this local practice of eyelash epilation in the absence of TT (as demonstrated by our 2013 prevalence survey data)[14] had a significant impact on the 2012 estimate of TT prevalence in Western Division. Our adjusted prevalence estimate of regular epilation in Western Division (8.6% of those aged ≥15 years) is very similar to the 2012 TT prevalence estimate (8.7%) in the same population^2^. This may account for the apparent discrepancy between TT prevalence estimates (8.7% in 2012; 0% in 2013) and the lack of people presenting locally for TT surgery. Two individuals with TT reportedly presented to surgical outreach clinics in Fiji between 2011 and 2013, neither of whom received corrective surgery[23].

The origin of the epilation behaviour in this population is unclear, though our data help to generate hypotheses and exclude some potential explanations. The most commonly described symptom motivating epilation was itchiness, whereas trichiasis is more commonly associated with painful, watery or purulent eyes, or blepharospasm[6]. Neither the natural history nor the examination findings in our subjects were consistent with other ocular pathologies that can lead to trichiasis, such as involutional changes linked to senescence, marginal entropion from chronic inflammation due to blepharitis or meibomian gland disease, or distichiasis, when an extra row of maldirected eyelashes is present[24]. These are all conditions that may prompt individuals to epilate, but are considered rare at population level. A psychological cause (such as the impulse control disorder trichotillomania, whereby the individual cannot control urges to pull out their own hair) was inconsistent with the descriptions given by the focus group.

Drinking of kava, and particularly drinking kava often, was a strong independent risk factor for regular epilation in our population. Kava (*Piper methysticum*) is a perennial plant that can be used to prepare a non-alcoholic drink by mixing the ground root and stem bases with water[25]. Kava has been used in South Pacific communities for centuries, for medicinal, social and cultural purposes[26]. The active ingredients are kavalactones, which are associated with eye itchiness as a side effect. Participants in the focus group felt that consumption directly precipitated eye symptoms; others felt symptoms could be brought on by sleep deprivation associated with its use. In a randomised controlled trial in Tonga, kava drinkers were reported to have “red, irritated eyes and increased photosensitivity during periods of heavy drinking”[27].

It seems likely that kava may produce eye symptoms, but the potential mechanism for relief through epilation is unclear. It is possible that it could be a form of distraction, either psychologically, or physically through the pain commonly associated with epilation. Importantly, although drinking kava was strongly associated with epilation, 56 (45%) of the 125 epilators reported never drinking kava. This supports the idea that in Fijian custom a variety of eye symptoms might be considered to be amenable to epilation, with itchiness from kava drinking being just one. It is possible that epilation in this context could also represent a cultural practice reflective of a time when trichiasis was more common and that this persisted even after trichiasis became rare.

As further evidence of a cultural determinant of this behaviour, in the full model, we found that iTaukei individuals had 6.0 times greater odds of being an epilator than those of any other ethnicity. The major ethnic groups in Fiji are iTaukei and Indo-Fijian—both with distinct cultures, practices, beliefs and languages. iTaukei are predominantly Christian and speak an indigenous language, whereas Indo-Fijians are mainly Hindu or Muslim and speak a local variant of Hindi. The overall population of Fiji comprises 56.8% iTaukei and 37.5% Indo-Fijian, with the remainder being a mixture of European, Chinese, Rotuman and other Pacific Islanders.[11]

Although its origins are elusive, significant differences are seen between the epilation behaviour described here, and the anticipated behaviour if epilation was related to trichiasis. Both the number of eyelashes removed and the frequency of epilation found in this population are noticeably different from those normally reported in the context of TT. A study in Ethiopia, for example, found that among individuals with trichiasis who self-managed symptoms by epilating, there was a median of 2 eyelashes touching the eye (interquartile range 1–5),[9] whereas in our population, the majority of epilators (66%) removed more than 10 eyelashes each time. In addition, the same Ethiopian study found that 96% epilated at least once per month, with 51% epilating at least once per week,[9] consistent with the need to relieve symptoms from eyelash regrowth, whereas in Fiji we found only 16% of epilators epilated at least once per month, with only 3% epilating at least once per week.

As is commonly found in household surveys carried out during the day, we have an under-representation of males in the survey. As only those present at the household at the time of survey were enumerated (rather than all household inhabitants whether present or not), it is not possible to determine the true extent to which men were under-represented in the households sampled. It is possible that, given that both survey field researchers were female, there might have been higher uptake from female community members. This could have potentially been addressed with a stronger approach to community sensitisation in advance of the surveys. Our focus group discussion took place in an iTaukei village because when it was conducted we had not heard that epilation was also practised by other ethnic groups; it is possible that this influenced the choice of possible risk factors assessed at population level. A further potential weakness of our work is the inherent difficulty in standardising the diagnosis of TS, which was not a feature of the training and standardisation of graders carried out as part of the Global Trachoma Mapping Project[16]. We tried to compensate for this last weakness by taking conjunctival photographs and having the images reviewed by highly experienced trachoma researchers—though even here, one image (Fig 2) was controversial.

In this study we have highlighted an epilation behaviour that could significantly bias TT prevalence estimates. We conclude that epilation in the absence of trichiasis has the potential to impact programme planning for trachoma elimination in countries where this practice is common. Further studies in Pacific populations where kava is consumed should be undertaken to see whether the relationship with kava drinking seen in our study is true in other settings. Given the difficulties inherent in determining whether epilated eyelashes were in-turned, consideration should be given in future surveys to require the presence of TS in epilated eyelids in order to diagnose TT.

## Supporting Information

S1 Appendix Questions used by the focus group moderator to guide discussion about existing knowledge of trichiasis and epilation, and the causes and natural history of common local eye complaints, Western Division, Fiji, 2013. (DOCX)

S2 Appendix Data collected in the questionnaire by the survey team, Western Division, Fiji, 2015. (DOCX)

S1 Checklist STROBE checklist for reports of observational studies. (DOCX)
