# Supplementary material for: Eyelash Epilation in the Absence of Trichiasis: Results of a Population-Based Prevalence Survey in the Western Division of Fiji
Source: PLoS Negl Trop Dis. 2017 Jan 23;11(1):e0005277. doi: 10.1371/journal.pntd.0005277 (PMC5256864; doi:10.1371/journal.pntd.0005277)
Supplement: S1 Appendix — (DOCX) [file pntd.0005277.s001.docx]

**Appendix 1: Questions used by the focus group moderator to guide discussion about existing knowledge of trichiasis and epilation, and the causes and natural history of common local eye complaints, Western Division, Fiji, 2013.**

1. What would make people think there was something wrong with their eyes?
2. What would make someone think they should see a doctor about their eye problem?
3. Could people describe why they think they pluck their eyelashes?
4. Has anyone ever had eyelashes that curl round and touch the front of their eye?
5. Is anyone surprised that people don’t tend to do this in other countries?
6. Does anyone have any thoughts on why only Fijians might do this?
   1. Rarely indo-fijians, but although globally.
7. What general advice do friends and family give when you have a problem with your eyes?
8. How do you pluck your eyelashes?
   1. Threading/tweezers/coconut husks/fingers
9. How many eyelashes would you pluck?
10. Has anyone every pluck out all of their eyelashes?
11. How soon after plucking their eyelashes would people expect to feel relief?
12. How often do people do it?
    1. Every day? Once/week? Once/month?
13. It is related to time of day?
    1. Morning/night/just after work/kava?
14. What jobs do people have?
15. Where do you come from?
16. Did you move here or have you always lived here?
17. When did you start plucking your eyes?
18. How did you learn how to do it?
19. What makes you want to do it?
20. Does plucking your eyelashes relieve the reason you do it?
21. Are there other reasons that people pluck their eyelashes?
22. Is it related to nervousness?
    1. Does anyone bite their nails?
    2. Pull eyebrow hairs?
23. Some people describe it as an itchiness. Is this what people think?
24. Has anyone had an eye itchiness and watery eyes that was in both their eyes?
    1. Did plucking eyelashes help with this?
25. Does anyone think it is related to vanity/wanting to look nice?
