# Supplementary material for: Eyelash Epilation in the Absence of Trichiasis: Results of a Population-Based Prevalence Survey in the Western Division of Fiji
Source: PLoS Negl Trop Dis. 2017 Jan 23;11(1):e0005277. doi: 10.1371/journal.pntd.0005277 (PMC5256864; doi:10.1371/journal.pntd.0005277)
Supplement: S2 Appendix — (DOCX) [file pntd.0005277.s002.docx]

**Appendix 2: Data collected in the questionnaire by the survey team, Western Division, Fiji, 2015.**

| Age | NUMERICAL |
| --- | --- |
| Gender | MALE/FEMALE |
| Ethnic group | ITAUKEI/INDO-FIJIAN/OTHER |
| Do you drink kava? | YES/NO |
| IF YES – How frequently do you drink kava? | DAILY/WEEKLY/MONTHLY/LESS THAN MONTHLY |
| Do you have a history of eye problems? | YES/NO |
| IF YES – Record reported problem | |
| In the last week have you experienced: | |
| Eye itchiness | YES/NO |
| Eye redness | YES/NO |
| Eye discharge/crusting | YES/NO |
| How often do you epilate your eyelashes? | NEVER/AT LEAST ONCE A WEEK/BETWEEN ONCE A WEEK AND ONCE A MONTH/EVERY 1-3 MONTHS/GREATER THAN EVERY 3 MONTHS |
| **If Individual Reports Epilation: Complete the following section** | |
| When you epilate how many lashes do you typically remove? | 1 LASH/2-10 LASHES/>10 LASHES/MOST OR ALL OF MY LASHES |
| Please rate each factor on how it affects your decision to epilate:  1: Not at all important  3: Somewhat important  5: Very important | |
| Itching sensation | 1-5 |
| Cosmetic reasons | 1-5 |
| Feeling of dust in eye | 1-5 |
| Prolonged sun exposure | 1-5 |
| Habit | 1-5 |
| Tradition | 1-5 |
| Eyelash appears abnormal | 1-5 |
| At approximately what age did you first epilate? | NUMERICAL |
| **OCULAR EXAMINATION** | |
| Is there evidence of trichiasis? | YES/NO |
| Is there evidence of trachomatous scarring? | YES/NO |
